# The *Cryptococcus neoformans* capsule: lessons from the use of optical tweezers and other biophysical tools

**DOI:** 10.3389/fmicb.2015.00640

**Published:** 2015-06-24

**Authors:** Bruno Pontes, Susana Frases

**Affiliations:** ^1^Laboratório de Pinças Óticas da Coordenação de Programas de Estudos Avançados, Instituto de Ciências Biomédicas, Universidade Federal do Rio de Janeiro, Rio de Janeiro, Brazil; ^2^Laboratório de Ultraestrutura Celular Hertha Meyer, Instituto de Biofísica Carlos Chagas Filho, Universidade Federal do Rio de Janeiro, Rio de Janeiro, Brazil

**Keywords:** *Cryptococcus neoformans*, polysaccharide, capsule, optical tweezers, light scattering, viscosity, zeta potential

## Abstract

The fungal pathogen *Cryptococcus neoformans* causes life-threatening infections in immunocompromised individuals, representing one of the leading causes of morbidity and mortality in AIDS patients. The main virulence factor of *C. neoformans* is the polysaccharide capsule; however, many fundamental aspects of capsule structure and function remain poorly understood. Recently, important capsule properties were uncovered using optical tweezers and other biophysical techniques, including dynamic and static light scattering, zeta potential and viscosity analysis. This review provides an overview of the latest findings in this emerging field, explaining the impact of these findings on our understanding of *C. neoformans* biology and resistance to host immune defenses.

## Introduction

The basidiomycete fungus *Cryptococcus neoformans* is an opportunistic human pathogen that causes cryptococcosis, a life-threatening disease that kills over 600,000 people a year (Arturo and Perfect, 1998). Cryptococcosis is one of the leading causes of morbidity and mortality among AIDS patients (Park et al., 2009); however, some cases have also been documented in immunologically competent individuals (Rozenbaum and Goncalves, 1994; Chen et al., 2000; Pappas et al., 2001). Although the disease is a worldwide threat, underdeveloped countries experience particularly high mortality rates. Current antifungal-based therapies against cryptococcosis are often incapable of completely eliminating the pathogen, leading to the recurrence of the disease (Bicanic and Harrison, 2004). Thus, the development of novel therapeutic strategies is paramount, but depends on an improved understanding of *C. neoformans* biology and pathogenesis.

Several virulence factors allow *C. neoformans* to evade host defense mechanisms, enabling pathogen survival and dissemination in the host tissues (Casadevall and Pirofski, 2007). While virulence factors include melanin production (Huffnagle et al., 1995; Wang et al., 1995; Doering et al., 1999; Liu et al., 1999; Gomez and Nosanchuk, 2003), phenotypic switching (Goldman et al., 1998; Fries et al., 2001), and cellular “gigantism” (Okagaki et al., 2010; Zaragoza et al., 2010), the most significant and hazardous feature of *C. neoformans* is the ability to produce a polysaccharide (PS) capsule, which represents the main virulence factor of this fungal species. The capsule protects the fungal cell against a variety of host immune defenses (Park et al., 2009), and plays diverse roles in the development of cryptococcosis.

Cryptococcal PS can be secreted via the protein secretory pathway (Yoneda and Doering, 2006; Panepinto et al., 2009), or shed from cells in association with vesicular structures (Rodrigues et al., 2007), and consists of at least two types of polymers: glucuronoxylomannan (GXM) and glucuronoxylomannogalactan (GXMGal; Heiss et al., 2009). Despite the homogenous appearance of the capsule by light microscopy, electron microscopy (EM) data suggest that the capsule is a heterogeneous structure, with a clear vertical “stratification” of the matrix into an electron-dense inner layer and an electron-lucent outer layer (Gates et al., 2004; Bryan et al., 2005; Frases et al., 2009b). While the outer layer is more permeable, the inner layer of the capsule matrix prevents larger macromolecules—including antibodies and complement proteins—from reaching the cell wall (Gates et al., 2004; Gates and Kozel, 2006; Frases et al., 2009b), protecting the fungus from the deleterious effects of these immune system molecules.

Many fundamental aspects of capsule structure, mechanics and dynamics remain poorly understood, partly due to its complex composition and organization. Also, the capsule is sensitive to the dehydration and fixation procedures used in conventional EM, which limits severely the number of available methods for native PS structural characterization. Recently, the use of a variety of biophysical tools—including static and dynamic light scattering (DLS), as well as zeta potential, optical tweezers and PS viscosity analysis—has improved considerably our understanding of the macromolecular properties of the *C. neoformans* PS capsule, challenging earlier views on capsule architecture and assembly (McFadden et al., 2006; Nimrichter et al., 2007; Frases et al., 2008, 2009a,b; Cordero et al., 2011b, 2013; Araujo Gde et al., 2012).

This review focuses on the novel data on the biophysical properties of the capsule uncovered by the use of different tools, highlighting the correlation between these properties and capsule structure and function.

## Studying the Capsule PS Structure using Light Scattering, Zeta Potential and Viscosity Analysis

Novel and interesting macromolecular properties of the *C. neoformans* capsule PS, and the impact of these properties on PS biological activity, have been uncovered by combining concepts from the theory of polymer solutions with the use of a variety of biophysical techniques, including static and DLS, zeta potential and viscosity analysis.

Light scattering represents alterations—by reflection, refraction and diffraction—in the direction and intensity of a light beam after it interacts with particles. The magnitude of light scattering by a given macromolecule suspension depends on the size and concentration of the molecules, as well as on their “polarizability,” which reflects structural features of the macromolecules being analyzed. Polarizability is calculated using the differential index of refraction—the ratio between the refractive index of the molecules and that of the surrounding medium—and polarizability data obtained by light scattering analysis reveals important structural information on macromolecules in solution (Schärtl, 2007).

Static light scattering (SLS), which measures light scattering intensity at different angles, allows the determination of the weight-average molecular weight, the molecular size (or radius of gyration) and the second osmotic virial coefficient (Schärtl, 2007) of macromolecules in suspension. On the other hand, DLS measures fluctuations (over time) in the intensity of the light scattered by macromolecules diffusing in solution due to Brownian motion. These fluctuations are a direct result of macromolecular diffusion, which alters the otherwise monochromatic incident light, generating scattered waves of different wavelengths—a phenomenon known as the “Doppler effect.” If the temperature and solvent are constant and known, the time-dependent fluctuations in scattered light intensity (detected and analyzed using an autocorrelator) are directly related to molecular size, and can be used to calculate the average-hydrodynamic radius and “polydispersity” of macromolecules (Berne and Pecora, 2013).

Another powerful biophysical tool for the analysis of macromolecules in suspension is the zeta potential, defined as the difference between the electric potential of the dispersion medium and that of the stationary layer of fluid attached to dispersed particles. By applying an electric field to a macromolecule solution and monitoring the movement of dispersed macromolecules, zeta potential measurements can be obtained and used to assess the ability of molecules to either come together or remain stably dispersed, due to repulsive electric forces between them (Hunter et al., 2013). Importantly, zeta potential measurements allow for rapid estimation of relative differences in composition between ionic polymers.

Viscosity analysis—representing measurements of the resistance of a macromolecular fluid to flow—is also used to infer important properties of macromolecules. The viscosity of a solution can be obtained by the ratio of the applied shear stress to the resulting strain rate. The most common method for viscosity analysis consists of using a capillary viscometer (e.g., an Ostwald viscometer) submerged in a temperature-controlled water bath, to compare the flow times of polymer solutions of different concentrations with that of pure solvents (under constant temperature, pressure and volume). High molecular weight polymers have higher intrinsic viscosity than low molecular weight and/or linear polymers (Landel and Nielsen, 1993). Thus, viscosity measurements can be used to infer the molecular weight of polymers in solution.

Until recently, most of what was known on capsule properties had been inferred from data on secreted PS recovered from culture supernatants (Cherniak et al., 1988), believed to represent shed capsular material. However, a comparison of secreted PS isolated by two different techniques with capsular PS stripped from cells using either gamma radiation or dimethyl sulfoxide (DMSO) treatment revealed significant differences in glycosyl composition, mass, size, charge, viscosity, circular-dichroism spectra, and monoclonal antibody reactivity (Frases et al., 2008), strongly suggesting that secreted PS and capsular PS are structurally different, and questioning the use of secreted PS as a surrogate for the capsule PS, in biochemical studies. Also, these data confirm that the extraction/isolation method may influence significantly the structural and antigenic properties of PS fractions (Nimrichter et al., 2007).

Analysis of the size of capsule GXM molecules removed from cryptococcal cells by DMSO revealed a wide distribution, with average-molecular weights ranging from kilo- to mega-Daltons (McFadden et al., 2006, 2007). Also, these and other datasets highlight the considerable inter-strain variability in the properties of the cryptococcal capsular PS (Cherniak et al., 1988, 1995; McFadden et al., 2007). Macromolecular characterization of capsular PS from different cryptococcal strains shows that these molecules are particularly large, exhibiting weight-average molecular weights from 10^7^ to 10^8^ g mol^–1^, molecular sizes ranging from 158 to 239 nm, and average hydrodynamic radius values ranging from 570 to 2434 nm, as determined by static and DLS. The high values of average molecular weight observed for capsular PS molecules are consistent with previous reports (Frases et al., 2008), and confirm a fundamental difference between capsular and secreted PS molecules, since the latter has average molecular weights from 10^5^ to 10^6^ g mol^–1^ only (McFadden et al., 2006, 2007; Frases et al., 2008). To our knowledge, the cryptococcal capsular PS molecules are the largest PS molecules described to date.

Data derived from DLS and SLS measurements, such as the angular dependency and the relationship between the weight-average molecular weight and the molecular size—known as the “shape factor”—provide values consistent with the notion that the cryptococcal PS is branched (Cordero et al., 2011a). Moreover, the viscosity behavior of capsular PS at relatively low concentrations are consistent with branching, because they suggest a high degree of entanglement and inter-particle interaction in the absence of applied force (Cordero et al., 2011a). Also, isolated capsular PS molecules appear as rosette-like structures similar to those of other branched PS (e.g., glycogen), when visualized by EM (Childress et al., 1970; Tao et al., 2007). Thus, the EM data on isolated PS is in agreement with the light scattering data, and provide clear evidence that the cryptococcal capsule PS is branched.

Polysaccharide branching is not the norm in nature, since other microbes exhibit capsules composed of linear homopolymers formed in the extra-cytoplasmic environment (Whitfield and Roberts, 1999; Garcia et al., 2000; Garcia-Rivera et al., 2004; Whitfield, 2006; Yother, 2011). The ability of *C. neoformans* to synthesize complex heteropolymers that are both large and branched may stem from the fact that, contrary to bacterial capsular polymers, cryptococcal PS are generated in the intracellular environment and exported to the extracellular space via vesicle-mediate secretion (Feldmesser et al., 2001; Garcia-Rivera et al., 2004; Yoneda and Doering, 2006; Rodrigues et al., 2007). Thus, biochemical synthesis of modified branched sugars and/or cross-linking (either enzymatic or non-enzymatic) may occur as PS molecules migrate through distinct chemical environments within the cell, *en route* to the cell surface.

The impact of a branched conformation on cryptococcal PS activity was demonstrated by comparing capsular PS samples exhibiting different degrees of PS branching (based on their shape factor values), but equivalent glycosyl composition (Cordero et al., 2011a). These data suggest that the degree of branching and/or structural complexity influences the ability of capsular PS to interfere with phagocytosis, stimulate nitric oxide production by macrophages, and protect against ROS and antibody reactivity, and also affects the half-life of PS molecules in serum.

## Different Applications of Optical Tweezers in the Examination of Capsule Structure and Function

Laser-based optical trapping emerged in the early 1970s, when Arthur Ashkin demonstrated that optical forces could displace micrometer particles in water solutions (Ashkin, 1970). These seminal work led to the development of single-beam optical traps or “optical tweezers” (OT; Ashkin et al., 1986), which allow micromanipulation of cells and molecules using forces and displacements in the piconewton (pN) and nanometer (nm) ranges respectively, corresponding to the scales of important physical and biological events. Thus, OT represents an ideal technique to study biological phenomena in detail (Neuman and Block, 2004; Moffitt et al., 2008). OT applications range from the study of molecular motors at single-molecule level (Veigel and Schmidt, 2011; Elting and Spudich, 2012), to the determination of the mechanical properties of biopolymers (Greenleaf et al., 2007), and cellular structures (Pontes et al., 2008, 2011, 2013).

An OT is formed by focusing a laser beam onto a particle observed under a light microscope. The particle experiences forces due to the transfer of momentum from the incident light, and the resulting optical force components are a “scattering” force that pushes the particle in the direction of light propagation, and a “gradient” force (i.e., one that pushes dipole-like particles in a inhomogeneous electric field toward the field gradient; Neuman and Block, 2004) that pulls the particle toward the laser source. Stable optical trapping is achieved by sharply focusing the laser beam (using an objective of high numerical aperture) onto a particle near the focal point of the lens, where the gradient force pulling the particle toward focus exceeds the scattering force pushing it away from focus. The OT then acts as a Hookean spring, whose stiffness is proportional to the light intensity; since the displacement of a trapped particle can be measured by imaging techniques, the force producing the displacement can be determined easily following Hooke’s law. These force values can then be used to calculate important mechanical properties of polymers and cells.

Optical tweezers-based methods have emerged as useful tools in the study of the *C. neoformans* capsule, by allowing micromanipulation of the capsule in its native state, as described in the pioneering work of Frases et al. (2009a). Briefly, the capsule of live *C. neoformans* yeast cells is deformed with the aid of a polystyrene bead attached to the capsule and trapped in an OT, and the displacement of the bead’s center of mass is monitored as a function of time. Then, a stress-strain curve is produced and used to determine the elastic properties (also known as the “Young’s modulus”) of the *C. neoformans* capsule. The higher the Young’s modulus, the greater is the capsule rigidity. A major advantage of this method is that capsular mechanical properties can be probed with high accuracy and reproducibility, as well as under conditions were the cells remain alive. Moreover, the Young’s modulus represents a useful quantitative parameter to evaluate capsule architecture under different conditions. In fact, several applications of this method have already been reported (Frases et al., 2009a; Cordero et al., 2011b, 2013; Araujo Gde et al., 2012).

In its original description, the OT method described above was used to evaluate the effect of treatment with divalent cations on the PS capsule (Frases et al., 2009a). Divalent cations can induce the self-aggregation of *C. neoformans* PS fibers—possibly by forming intra- and/or intermolecular links between (or within) glucuronic acid residues—and this effect could contribute to maintain capsule architecture and PS aggregation (Nimrichter et al., 2007). Moreover, divalent cations can also modify important biological properties of the *C. neoformans* capsule, such as its antibody reactivity and electronegativity (Frases et al., 2008). Frases et al. (2009a) investigated the hypothesis that cation-mediated aggregates and non-aggregated *C. neoformans* capsules have different elastic properties, by incubating EDTA-pretreated yeast cells in media with different Ca^2+^ concentrations (0–20 mM). Young’s modulus values increased as a function of ion concentration, supporting the view that divalent cations create cross-links that stabilize capsular structure (Frases et al., 2009a). Thus, at higher Ca^2+^ concentrations, all cation-binding sites on PS molecules were presumably occupied, which led to the formation of a lattice of cross-linked fibers, explaining the significant increase in Young’s modulus values (Frases et al., 2009a).

The OT-based method for capsule analysis was also used to study capsule growth induced by cultivating *C. neoformans* in minimal media (Frases et al., 2009a). Examination of the elastic properties of the PS capsule over time in culture showed that cells with smaller capsules had higher Young’s modulus values than those with larger capsules (Frases et al., 2009a). These results are particularly interesting in light of earlier reports showing that variations in capsule size can influence fungal pathogenesis in animal models (Rivera et al., 1998). Moreover, the finding that larger capsules are more deformable correlates with the observation that the outer sections of the capsule are mainly formed by lower density PS molecules (Gates et al., 2004).

The effects of chronological aging (Bouklas and Fries, 2015) on the capsule was also analyzed using OTs (Cordero et al., 2011b). Senescent cells accumulate during the course of infection by *C. neoformans* (Jain et al., 2009), and cell aging has important implications during cryptococcosis, since the chronicity of this disease can be associated with the persistence of senescent cells in the lung (Goldman et al., 2000). To analyze changes in the elastic properties of the capsule throughout the aging process, Cordero et al. (2011b) performed elegant experiments where *C. neoformans* cultures that had reached stationary phase in capsule-inducing medium were inoculated into fresh medium, and the capsule Young’s modulus was determined every 12 h (for a total of 120 h) after inoculation (Cordero et al., 2011b). Initially, capsule Young’s modulus values decreased significantly (with lowest values obtained 24 h post-inoculation), followed by a progressive increase up to the values obtained before inoculation. Interestingly, the size of the capsule did not vary significantly during the experiments. These results suggest that the Young’s modulus of the *C. neoformans* capsule is a modular property that changes over time in culture (Cordero et al., 2011b).

Interestingly, clear alterations in capsule structure, as well as changes in capsule permeability, charge, and antigenic density, occurred during cell aging, and could explain the differences in Young’s modulus values (Cordero et al., 2011b). On average, capsules from “older” cells had PS molecules with reduced molecular size and average-hydrodynamic radius, and there was a decrease in the weight-average molecular weight distribution of polymer molecules during prolonged stationary phase growth. These results were unexpected, given that capsule enlargement during exponential growth was thought to be irreversible (Zaragoza et al., 2006), and no PS degrading enzymes have been described so far, while non-enzymatic acid hydrolysis of PS molecules is improbable (Cordero et al., 2011b). Interestingly, capsules from “older” cells contained α-1-3-glucans, structural components not previously observed in *C. neoformans* capsules (Cordero et al., 2011b). Overall, the report of Cordero et al. (2011b) strongly suggest that prolonged stationary phase growth triggers the degradation and remodeling of the *C. neoformans* capsule, and that the capsule is a highly dynamic structure capable of readily changing its physical, chemical and structural properties in response to external stimuli.

Although the molecular mechanisms behind capsule “aging” are unknown, this process is associated with important functional alterations, such as increased resistance to complement-mediated phagocytosis and antibody reactivity (Cordero et al., 2011b; Bouklas and Fries, 2015). The increased phagocytosis resistance of “older” capsules appears to be caused by altered complement deposition due to steric hindrance and decreased capsule permeability (Cordero et al., 2011b). In addition, the reduced electrostatic potential exhibited by “older” cells could contribute to this effect, as suggested by previous observations (Kozel et al., 1980).

A clinically relevant application of OT to the study of *C. neoformans* was the evaluation of capsule stiffness after incubation with protective or non-protective antibodies (Cordero et al., 2013), which may help explain some of the protective mechanisms of antibodies with therapeutic potential. Antibodies against GXM, the main capsule PS, mediate protection against *C. neoformans* infection in mice (Sanford et al., 1990; Mukherjee et al., 1992; Fleuridor et al., 1998), and have been tested for clinical use as therapeutic agents against cryptococcosis (Casadevall et al., 1998; Larsen et al., 2005). Protection seems to involve classical mechanisms, such as increased rates of phagocytosis, complement activation, and recruitment of inflammatory cells (Nussbaum et al., 1997; Yuan et al., 1998; Taborda and Casadevall, 2001; Taborda et al., 2003), as well as non-classical mechanisms, such as inhibition of PS release (Martinez et al., 2004), biofilm formation *in vitro* (Martinez and Casadevall, 2005) and changes in pathogen metabolism and gene expression (McClelland et al., 2010; McClelland and Casadevall, 2012). Using OT, Cordero et al. (2013) showed that binding of protective, but not non-protective, antibodies produced a concentration-dependent increase in capsule stiffness, likely due to antibody-mediated cross-linking of PS molecules. Increases in capsule stiffness may affect cell wall integrity sensors (Levin, 2005), which could trigger changes in gene expression, as well as PS release (Martinez et al., 2004) or biofilm formation (Martinez and Casadevall, 2005). Protective antibody binding also led to “trapping” of daughter cells in a sac-like structure derived from the parental cell’s capsule (Cordero et al., 2013). This defect in daughter cell formation/separation is expected to lead to reduced pathogen dissemination and increased chances of pathogen phagocytosis, in the context of infection.

## Conclusions and Perspectives

Biophysical techniques have proven to be powerful alternatives for the analysis of fundamental *C. neoformans* capsule properties, and have successfully expanded our knowledge on the synthesis, regulation, and function of this key virulence factor. In particular, OT has emerged as an important tool in the study of the biophysical properties of the PS capsule, especially because OT measurements provide information on the capsule in its native state, without the need to isolate capsule PS. Importantly, OT experiments provided basic information that could be useful in the design and development of capsule-targeted therapeutic strategies against *C. neoformans*. The same techniques may be informative if applied to the study of other encapsulated pathogens.

Nevertheless, the biophysical properties detailed above remain difficult to reconcile with the organization of capsular PS molecules as observed in EM images, where capsules often appears as a layers of dispersed long fibers with dimensions inconsistent with light scattering data (Frases et al., 2009b). These inconsistencies suggest that the fiber-like structures observed using current EM techniques most likely represent artifacts in capsule morphology that results from dehydration and collapse of adjacent capsular PS molecules. New EM techniques under development should allow ultrastructural analysis without the need for fixation, dehydration and critical point-drying steps that severely affect capsule preservation for EM. Furthermore, the use of atomic force microscopy (AFM), both as imaging technique and/or as force spectroscopy technique, may contribute to our knowledge of the PS capsule. In a near future, it is likely that the association of OT, in particular, with modern EM and AFM techniques will improve considerably our understanding of the structure of the *C. neoformans* capsule.

### Conflict of Interest Statement

The authors declare that the research was conducted in the absence of any commercial or financial relationships that could be construed as a potential conflict of interest.
